# Supplementary material for: G‐Quadruplexes act as sequence‐dependent protein chaperones
Source: EMBO Rep. 2020 Sep 18;21(10):e49735. doi: 10.15252/embr.201949735 (PMC7534610; doi:10.15252/embr.201949735)
Supplement: Supplementary file 1 — Appendix [file EMBR-21-e49735-s001.pdf]

Appendix file for Begeman et al.

Contents

Appendix Figure S1…………………………………………………………………………2

Appendix Figure S2…………………………………………………………………………3

Appendix Figure S3…………………………………………………………………………4

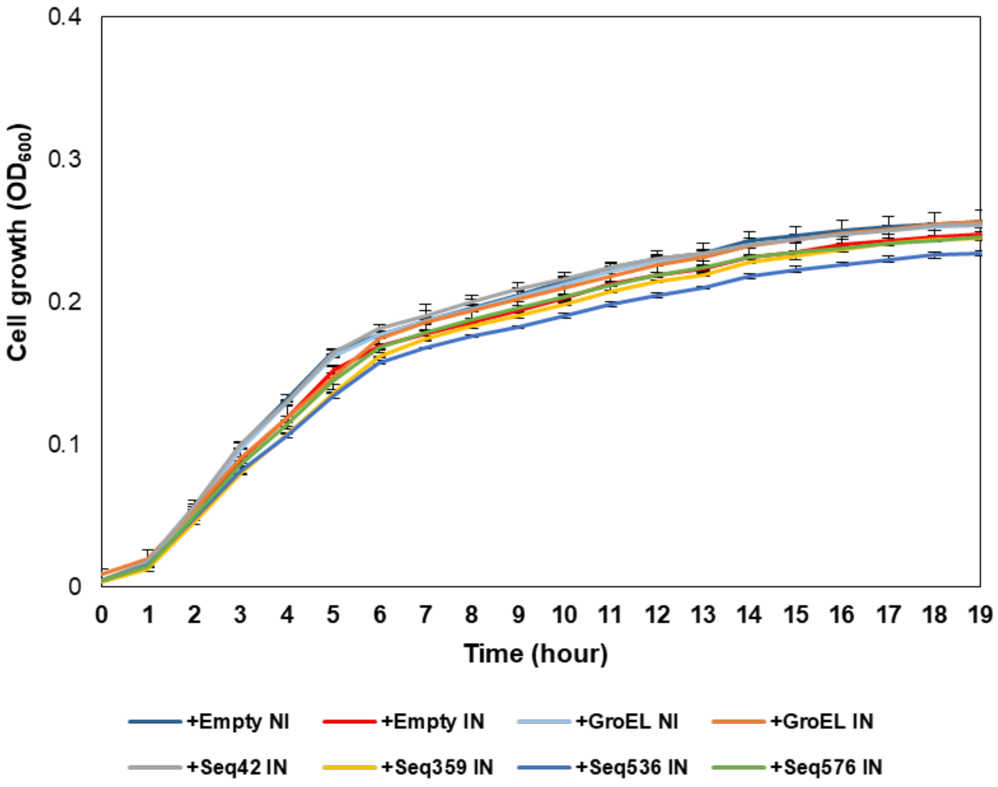

Appendix Fig S1. Growth curves of *E. coli* MC4100(DE3) in the presence or absence of G-quadruplex-containing sequences.

Absorbance at 600nm of cultured E. coli cells was measured for 19 hours, with the induction of GroEL, Seq42, Seq359, Seq536 and Seq576. Non-induced (NI) and induced (IN) Empty vector and non-induced GroEL were used as negative controls. The experiment was performed in triplicate; error bars are mean ± SD of biological triplicates (n=3).

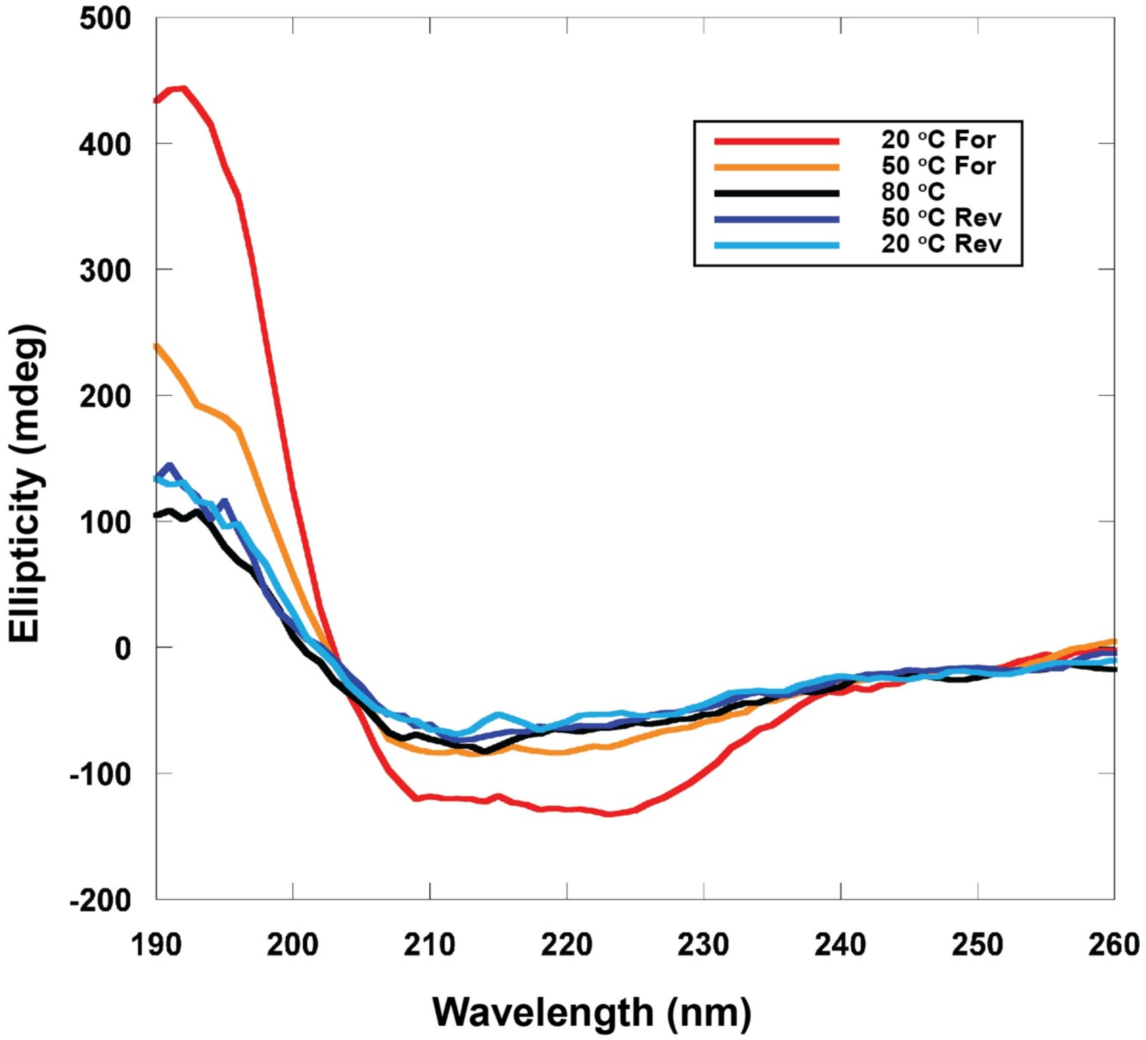

Appendix Fig S2. Circular dichroism of luciferase in the presence of quadruplex-forming sequence 359 throughout thermal denaturation.

Luciferase retains partial beta sheet structure even at extreme temperatures, which is retained after returning to lower temperature. "For" is the forward melt of the experiment while "Rev" indicates the reverse melting experiment.

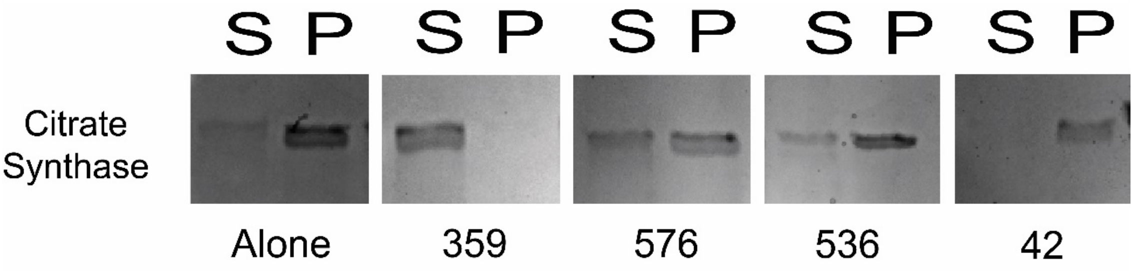

Appendix Fig S3. Prevention of protein aggregation in chemical spin down assay using citrate synthase.

Sequences 359, 536, and 576 all displayed holdase activity and contain a polyG motif. Sequence 42 was used as a negative control, as it performed poorly as a holdase chaperone and did not contain a polyG motif.
