# Supplementary material for: G‐Quadruplexes act as sequence‐dependent protein chaperones
Source: EMBO Rep. 2020 Sep 18;21(10):e49735. doi: 10.15252/embr.201949735 (PMC7534610; doi:10.15252/embr.201949735)
Supplement: Supplementary file 2 — Expanded View Figures PDF [file EMBR-21-e49735-s002.pdf]

Expanded View Figures

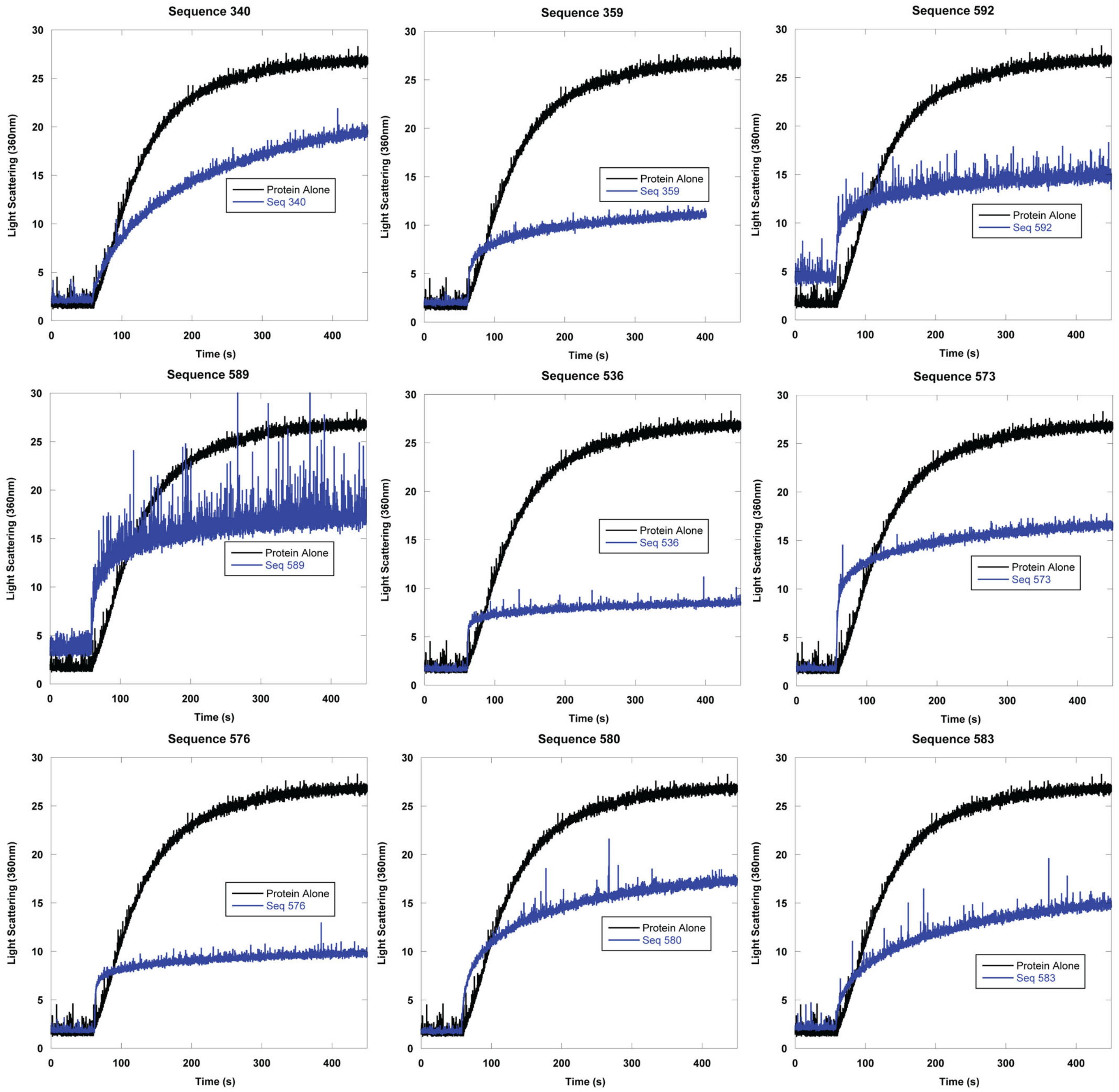

Figure EV1. Representative examples of chemical aggregation tests of multiple quadruplex-containing sequences with citrate synthase.

Representative examples of right angle light scattering of chemically denatured citrate synthase with quadruplex-containing sequences measured via fluorimeter. DNA strand to protein ratio is 2:1.

 © 2020 The Authors

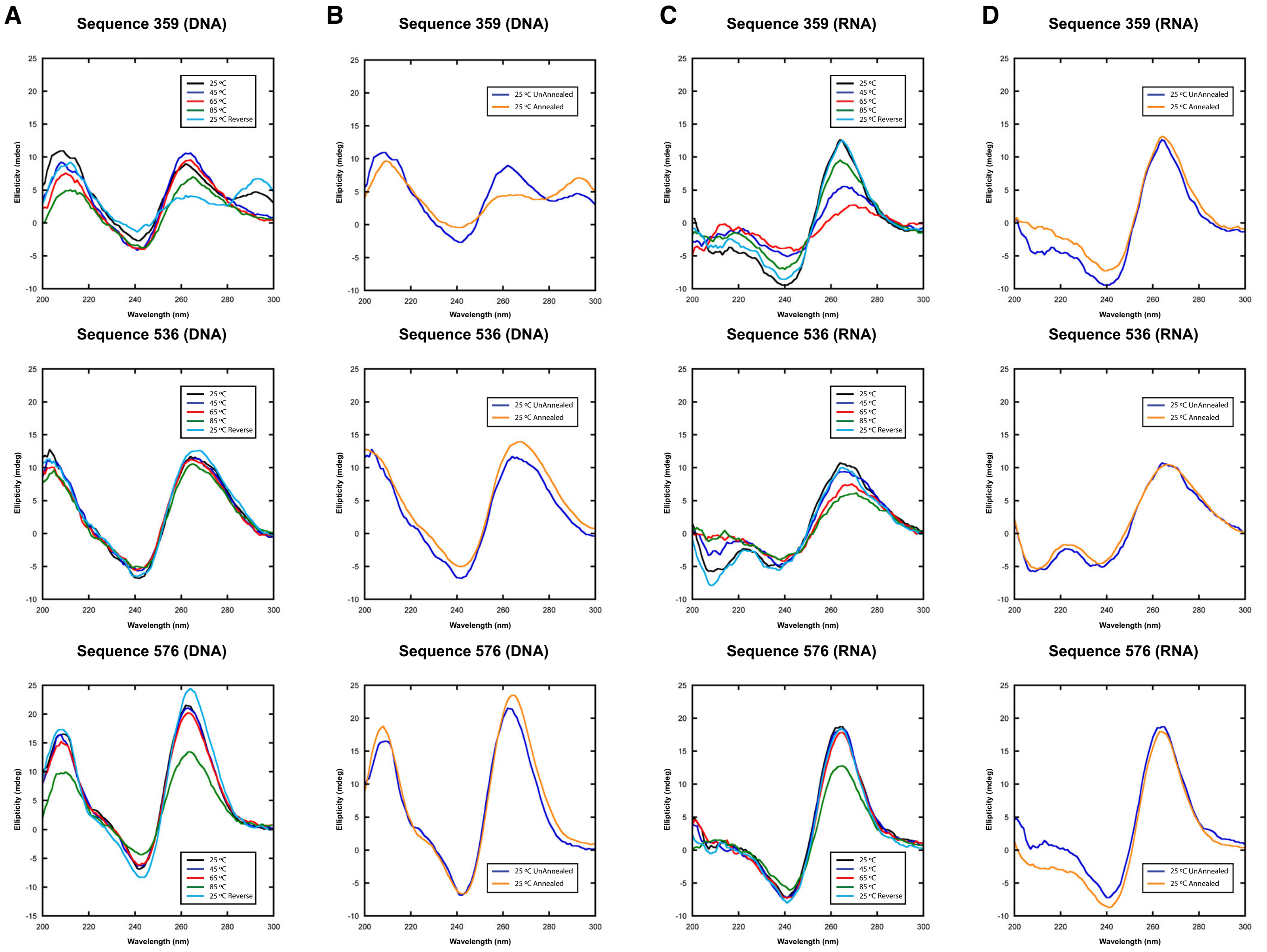

Figure EV2. CD spectra of select 20mers (DNA (A) and (B), and RNA (C) and (D)) in 10 mM pH 7.5 potassium phosphate.

The use of potassium phosphate here was to better mimic the initial aggregation screen in which potassium was used in the HEPES buffer.

A, C The thermal stability of quadruplex-containing sequences as measured by CD spectroscopy where each line represents a wavelength scan at the indicated temperature.

B, D The secondary structure of the same quadruplex-containing sequences at 25°C prior to annealing or after annealing at 25°C.

© 2020 The Authors

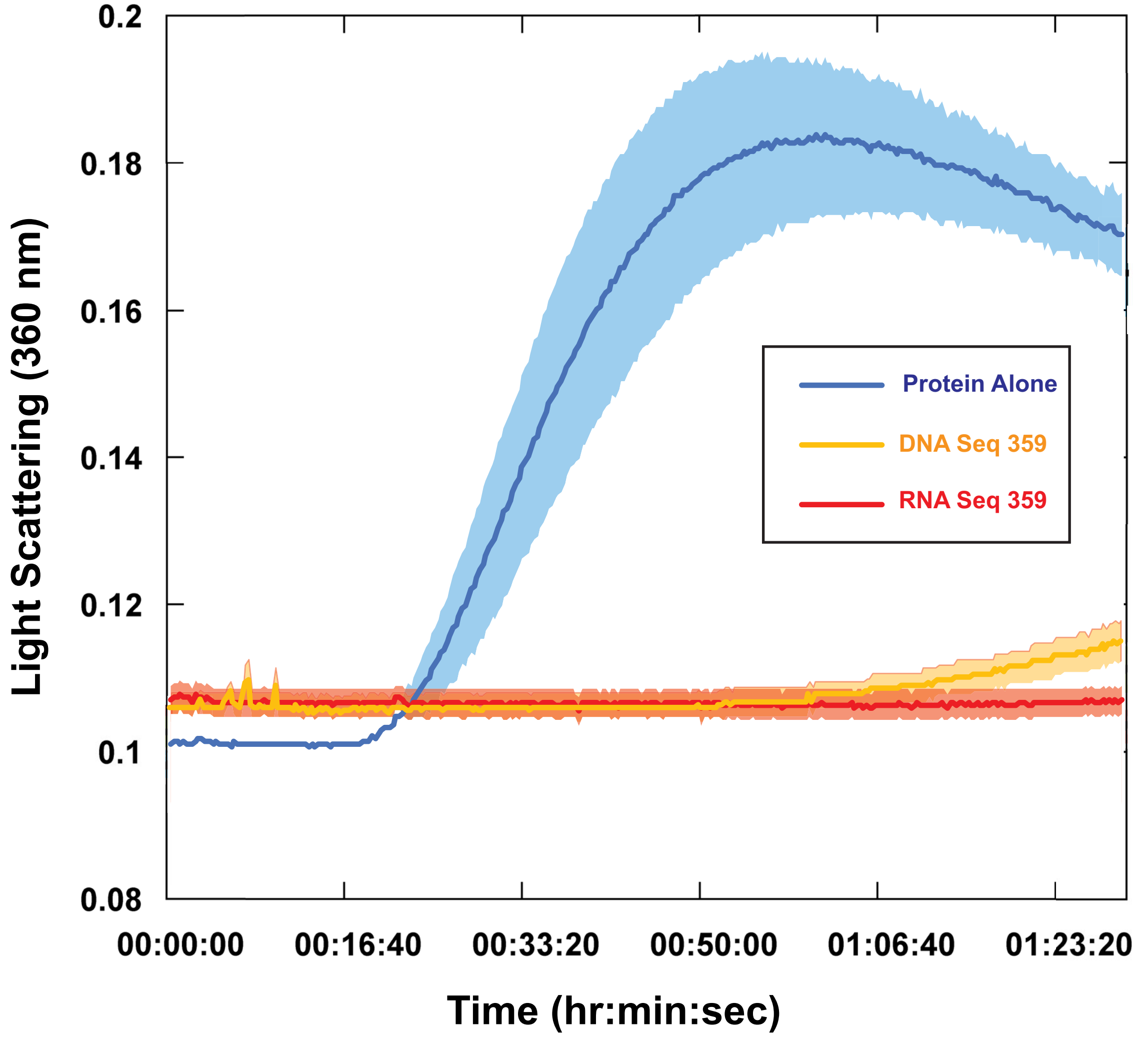

Figure EV3. Comparison of holdase activity of RNA and DNA counterparts of Sequence 359 using citrate synthase heat denaturation.

Data presented as means ± SE (n = 3) of technical triplicates.

Source data are available online for this figure.

 © 2020 The Authors

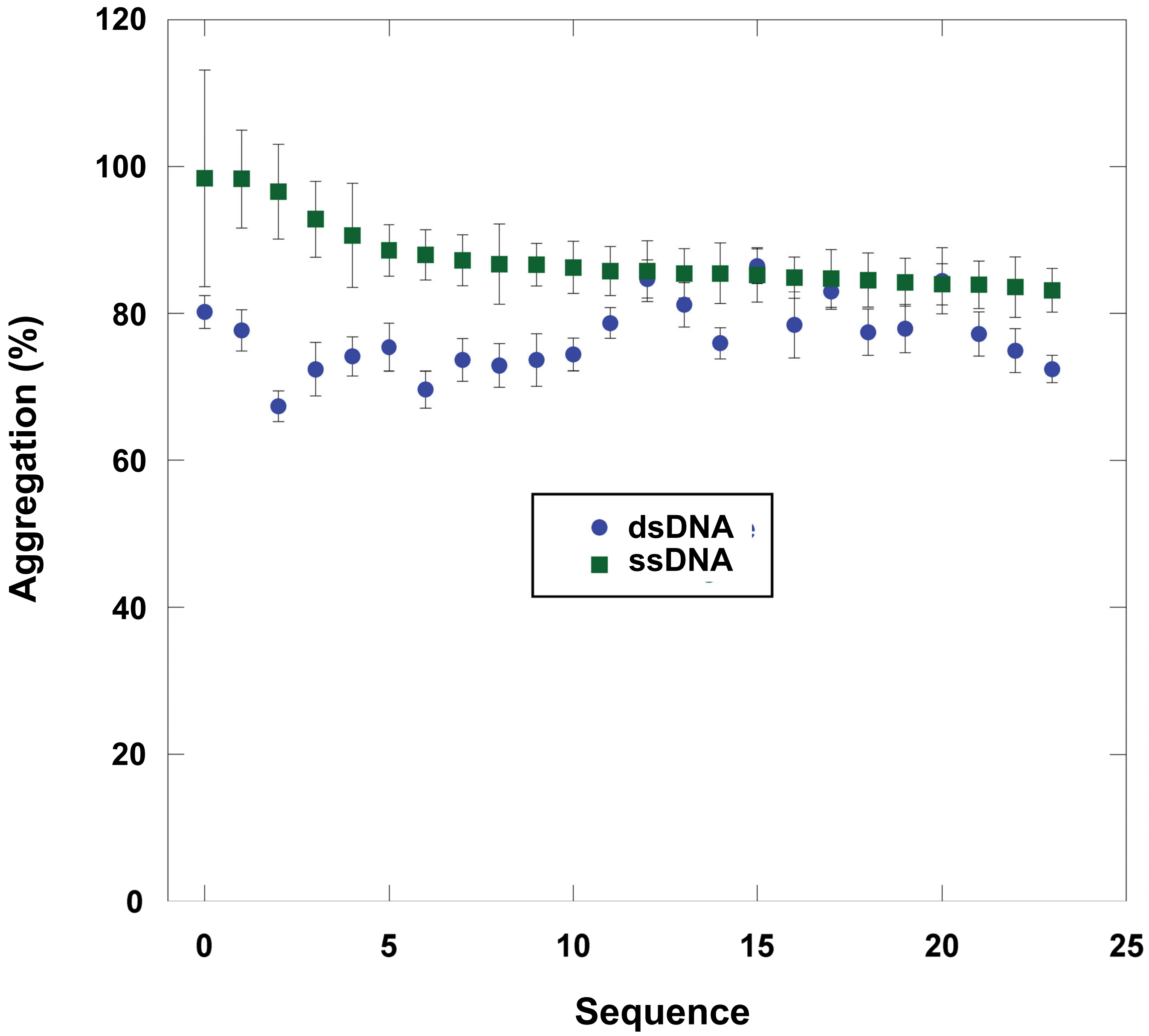

Figure EV4. Holdase activity of ssDNA toward citrate synthase compared to its duplexed counterpart as measured by heat denaturation.

Data presented as mean ± SE (n = 3) of technical triplicate.

Source data are available online for this figure.

Figure EV5. The effect of nucleic acids on the fluorescence of wtGFP.

A Expression vector constructs. Both vectors are under the control of pBAD promoter, which is induced by 0.2% *L*-Arabinose.

B Cellular fluorescence assay of wtGFP in the presence or absence of protein folding enhancing factors. White bars (+Empty IN and +Seq42) represent negative controls. The data are presented as mean ± SD (n = 3) of technical triplicate.

C *In vitro* refolding of wtGFP in the presence or absence of htDNA. GFP refolding curve assessing the fluorescence of GFP over a 60-minute incubation period, with denatured GFP both in the presence of htDNA (n = 6) and absence of htDNA (n = 6). Non-denatured GFP (Native) with htDNA (n = 4) was used as a control. Data are displayed as the mean of each experimental condition normalized to the blank with error bars represented as the Standard Error. All samples tested with technical replicates.

© 2020 The Authors

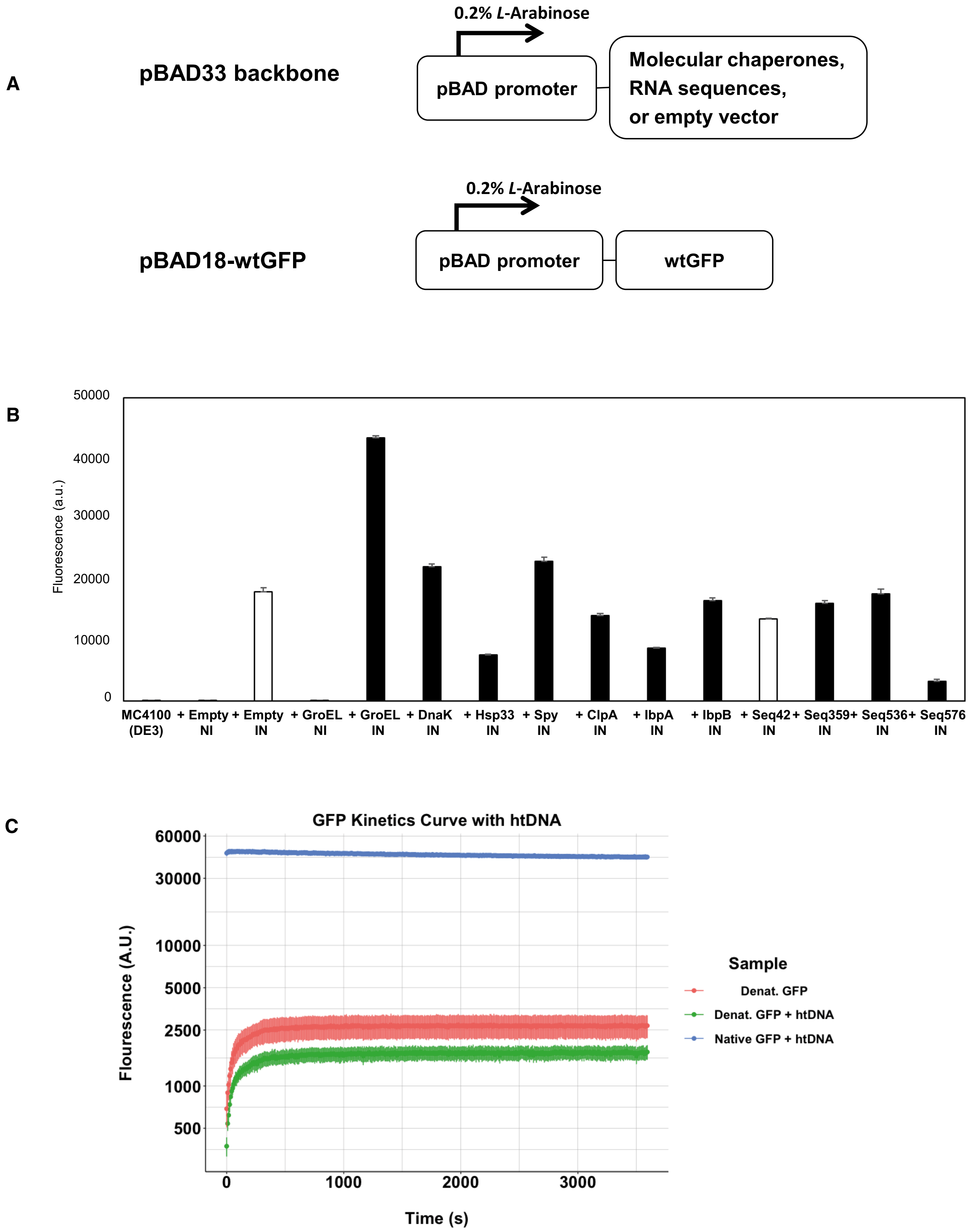

Figure EV5.

 © 2020 The Authors
