# Supplementary material for: G‐Quadruplexes act as sequence‐dependent protein chaperones
Source: EMBO Rep. 2020 Sep 18;21(10):e49735. doi: 10.15252/embr.201949735 (PMC7534610; doi:10.15252/embr.201949735)
Supplement: Supplementary file 3 — Table EV1 [file EMBR-21-e49735-s003.docx]

**Table EV1. Bacterial strains and plasmids**

| **Strain number** | **Strains** | | **Genotype** | | **References** |
| --- | --- | --- | --- | --- | --- |
| AS12 | MC4100(DE3) | | Δ*(argF-lac) U169 araD139 rpsLS50 reLAl deoCI ptsF25 rpsR flbB301* | | (Casadaban, 1976) |
| **Strain number** | **Plasmid1** | **Relevant Characteristics** | **Plasmid2** | **Relevant Characteristics** | **References** |
| AS181  AS197  AS171  AS183  AS185  AS187  AS189  AS191  AS193  AS195  AS173  AS175  AS177  AS179  AS199  AS209  AS211  AS212  AS213  AS214  AS215  AS216  AS201  AS203  AS205  AS207 | pBAD/HisD-TagRFP675  pBAD19-wtGFP  pBAD/HisD-TagRFP675  pBAD/HisD-TagRFP675  pBAD/HisD-TagRFP675  pBAD/HisD-TagRFP675  pBAD/HisD-TagRFP675  pBAD/HisD-TagRFP675  pBAD/HisD-TagRFP675  pBAD/HisD-TagRFP675  pBAD/HisD-TagRFP675  pBAD/HisD-TagRFP675  pBAD/HisD-TagRFP675  pBAD/HisD-TagRFP675  pBAD19-wtGFP  pBAD19-wtGFP  pBAD19-wtGFP  pBAD19-wtGFP  pBAD19-wtGFP  pBAD19-wtGFP  pBAD19-wtGFP  pBAD19-wtGFP  pBAD19-wtGFP  pBAD19-wtGFP  pBAD19-wtGFP  pBAD19-wtGFP | ApR  ApR  ApR  ApR  ApR  ApR  ApR  ApR  ApR  ApR  ApR  ApR  ApR  ApR  ApR  ApR  ApR  ApR  ApR  ApR  ApR  ApR  ApR  ApR  ApR  ApR | pBAD33mut-Empty  pBAD33-GroEL  pBAD33-DnaK  pBAD33-Hsp33  pBAD33-ClpA  pBAD33-Spy  pBAD33-IbpA  pBAD33-IbpB  pBAD33mut-Seq42  pBAD33mut-Seq359  pBAD33mut-Seq536  pBAD33mut-Seq576  pBAD33mut-Empty  pBAD33-GroEL  pBAD33-DnaK  pBAD33-Hsp33  pBAD33-ClpA  pBAD33-Spy  pBAD33-IbpA  pBAD33-IbpB  pBAD33mut-Seq42  pBAD33mut-Seq359  pBAD33mut-Seq536  pBAD33mut-Seq576 | CmR  CmR  CmR  CmR  CmR  CmR  CmR  CmR  CmR  CmR  CmR  CmR  CmR  CmR  CmR  CmR  CmR  CmR  CmR  CmR  CmR  CmR  CmR  CmR | (Piatkevich et al., 2013)  (Piatkevich et al., 2013)  This study  This study  This study  This study  This study  This study  This study  This study  This study  This study  This study  This study  This study  This study  This study  This study  This study  This study  This study  This study  This study  This study  This study  This study |
